# Assessment of Antibiotic Resistance and Microbial Contamination in Commercial Veterinary Probiotic Products

**DOI:** 10.3390/biology14111612

**Published:** 2025-11-17

**Authors:** Shuo Guan, Chunguang Wang, Zongshu Zhang, Mengfan Wang, Xinghua Zhao, Tie Zhang

**Affiliations:** College of Veterinary Medicine, Hebei Agricultural University, Baoding 071001, China; guanshuo218@163.com (S.G.); wangchunguang@hebau.edu.cn (C.W.); zs18233527535@163.com (Z.Z.); mengfan202312@163.com (M.W.); dyzhaoxh@hebau.edu.cn (X.Z.)

**Keywords:** antibiotic resistance genes, *Bacillus*, mobile genetic elements, co-occurrence network, probiotic quality control

## Abstract

Probiotics are widely used as feed additives in livestock production, yet the overall safety of commercially available veterinary probiotics remains insufficiently assessed. In this study, 33 probiotic products marketed in Northern China were systematically evaluated with respect to strain composition, label accuracy, antimicrobial resistance, and the diversity of resistance genes. A total of 32 *Bacillus* spp. were isolated, many of which showed resistance to multiple antibiotics. Labeling inaccuracies were prevalent: none of the products specified strain names and numbers, 33% (11/33) failed to report viable bacterial counts, 9% (3/33) lacked their claimed key ingredients, and 21% (7/33) contained isolated strains that did not match the label. High-throughput quantitative PCR (HT-qPCR) analysis further revealed that all 27 tested products harbored abundant antibiotic resistance genes (ARGs), with 241 ARGs and seven mobile genetic elements (MGEs) detected. The ARGs were primarily associated with tetracycline, aminoglycosides, β-lactams, and macrolide–lincosamide–streptomycin B (MLSB) antibiotics, and co-occurrence analysis showed a strong positive correlation between ARG and MGE abundance, with *Clostridium* and *Enterococcus* identified as potential hosts. These findings underscore significant quality and safety deficiencies in veterinary probiotics and highlight potential risks to animal, human, and environmental health, emphasizing the relevance of a One Health perspective in probiotic evaluation and regulation.

## Simple Summary

The use of antibiotics in animal farming has long raised concerns about drug residue and bacterial resistance, leading many countries to limit their use. As a result, probiotics are increasingly added to livestock feed as a natural alternative to improve animal growth and health. However, the safety of many commercial veterinary probiotics has not been properly assessed. In this study, we examined 33 probiotic products sold in Northern China to evaluate their quality and potential risks. We found that many products had inaccurate labels or missing information, and several contained bacteria resistant to multiple antibiotics. Genetic testing also revealed that nearly all products carried numerous antibiotic resistance genes that could spread to other microbes through genetic exchange. These findings indicate that some probiotic products may pose hidden safety risks to animals, humans, and the environment, highlighting the importance of a One Health perspective in the evaluation and regulation of veterinary probiotics. Strengthening quality control and safety assessment is essential to ensure their responsible use and support sustainable livestock production.

## 1. Introduction

Since long-term use of antibiotics can easily generate drug-resistant bacteria and cause harmful residues in animal products and other effects, banning the use of feed antibiotics in animal production has become a global consensus and an inevitable trend. Since the European Commission banned using antibiotics as feed additives in 2006 [1], many countries, including China, have also actively taken measures to restrict the application of antibiotics in livestock husbandry [2,3]. Thus, probiotic products for animal use have received increasing interest in both scientific and commercial fields. Probiotics are live microorganisms that provide a health benefit to the host when ingested in sufficient amounts [4]. Studies have shown that probiotic products for animal use can significantly improve the growth performance, immune function, and health status of animals in livestock and aquaculture industries [5,6]. The widespread use of veterinary probiotic products has also raised concerns about their quality and safety. In line with the One Health framework [7], rigorous safety assessment of veterinary probiotic products is essential because contaminated or mischaracterized formulations can compromise animal health, promote the transfer of pathogens or antimicrobial-resistance determinants to humans, and threaten environmental and food-chain safety.

Typically, veterinary probiotics consist of one or more selected different strains. In recent decades, *Bacillus* spp. have been widely utilized in animal probiotic products for their spore-forming ability, enabling long-term storage without the loss of viability. In addition, the spores can survive in the harsh, low-pH gastric environment and reach the animal intestine to exert their probiotic properties [8,9]. Several issues arose with the growth of the probiotic products market, such as incorrect labeling, inconsistent quality, and microbial contamination [10]. A study assessing the label accuracy of 25 commercial probiotic products found that only two product labels were compliant, with most veterinary probiotic products having defective labels [11]. Similarly, a previous study found that among 92 probiotic products available for animal use, over 1/3 were inaccurately labeled and contaminated with pathogens [8].

Compared to the issue of probiotic product label accuracy, microbial contamination and the carriage of antibiotic resistance genes (ARGs) in probiotic products are more serious. China, like many European countries, has specified the usable microorganisms in the “Feed Additives Catalog (2013)” [12]. Previous studies have reported that the extensive use of antibiotics in livestock farming promotes the accumulation of antimicrobial resistance (AMR) in animal waste and gut microbiota [13,14]. Furthermore, ARGs originating from animal production systems can spread to the surrounding soil, water, and air, contributing to environmental dissemination of resistance determinants [15,16,17]. In addition, horizontal gene transfer (HGT) mediated by bacteriophages, plasmids, and other mobile genetic elements (MGEs) has been recognized as a key mechanism facilitating ARG exchange among bacteria [18,19]. Building upon these previous findings, the present study investigates the occurrence of ARGs and MGEs in commercial veterinary probiotic products, aiming to assess their potential role as vectors of resistance dissemination under the One Health framework.

Thus, this study aims to conduct safety evaluations of commercial animal-use probiotic products and comprehensively investigate ARGs in probiotic products. Furthermore, the study reveals the issues and risks present in commercial veterinary probiotics, providing a scientific basis for the rational use of probiotic products in livestock husbandry, creating a favorable breeding environment, and promoting the sustainable development of animal husbandry.

## 2. Materials and Methods

### 2.1. Sample Sequencing and Sequence Analysis

A total of 33 probiotic products were obtained from 13 provinces/municipalities/autonomous regions (P/M/A) across China between 2023 and 2024 (Table 1). Within each region, products were randomly purchased from licensed veterinary drug distributors and feed suppliers to ensure compliance with national regulations and reduce potential selection bias. All products were labeled as probiotic preparations intended for livestock or poultry use. All probiotic products were stored according to the manufacturers’ recommended conditions and processed immediately after opening. The study evaluated the accuracy of probiotic product labels based on three aspects: whether the strain name is indicated on the label, whether the number of viable bacteria is described, and whether the main components identified on the label are isolated. The microbial composition of products was detected through sequencing of the V5–V7 region of bacterial 16S rRNA genes, with a size of 394 bp. Although the V5–V7 region has certain limitations in distinguishing closely related species and identifying functional genes compared with full-length 16S rRNA or metagenomic sequencing, it provides up to 92% accuracy at the genus level, offering a balanced trade-off between sequencing read-length constraints and taxonomic resolution. DNA was extracted from pre-processed probiotic products by using an OMEGA Soil DNA Kit (Omega Bio-Tek, Norcross, GA, USA). The quantity of DNA was determined using a Nanodrop (Thermo Fisher Scientific, Wilmington, DE, USA). Amplification by PCR was performed using the specific primers targeting the V5–V7 region of bacterial 16S rRNA. The PCR reaction system (20 µL) consisted of 0.25 µL of Q5 high-fidelity DNA polymerase, 5 µL (5×) Reaction Buffer, 5 µL (5×) High GC Buffer, 1 µL of (10 µM) each upstream and downstream primer, 2 µL of (10 µM) dNTP, 2 µL of DNA template and 8.75 µL of ddH_2_O. The PCR reaction conditions were as follows: 98 °C pre-denaturation was implemented for 5 min, followed by 98 °C for 30 s, 53 °C annealing for 30 s, 72 °C extensions for 45 s, and 72 °C final extension for 5 min.

### 2.2. Isolation and Identification of Bacillus spp.

A mixture of 0.5 g or 500 µL of the probiotic product was prepared in 10 mL of phosphate-buffered saline (PBS), and 15 µL of the probiotic suspension was cultured in 3 mL brain heart infusion (BHI) broth and incubated at 37 °C for 7 h for pre-enrichment. Then the bacterial culture was inoculated on mannitol–egg yolk–polymyxin agar (MYP) and incubated at 37 °C for 24 h. After isolation, each single colony with distinct color and morphology was passaged onto fresh agar plates. Genomic DNA was extracted from the isolated bacteria by using a TIANamp Genomic DNA Kit (Tiangen Biotech, Beijing, China), and amplification of the 16S rRNA gene was performed by using the combination of universal bacterial primers 27F and 1492R. The amplified products were sent to Sangon Biotech (Shanghai) Co., Ltd. for sequencing, and the sequencing results were subjected to nucleotide blast at NCBI (https://blast.ncbi.nlm.nih.gov, accessed on 10 November 2024) for further identification of *Bacillus* spp. isolated from probiotic products.

### 2.3. Antimicrobial Susceptibility Test

The susceptibility of *Bacillus* spp. to 13 antimicrobial agents (penicillin, ampicillin, ceftazidime, imipenem, meropenem, tetracycline, gentamicin, amikacin, erythromycin, clindamycin, vancomycin, ciprofloxacin, and levofloxacin) was determined by using the disc diffusion method (Table S1). Briefly, bacterial suspensions were prepared in sterile 0.9% saline from 24 h cultures grown on tryptic soy agar (TSA) and adjusted to a turbidity equivalent to a 0.5 McFarland standard. The suspensions were uniformly spread on Mueller–Hinton agar plates using sterile cotton swabs. Antibiotic disks were then placed on the agar surface, and plates were incubated at 37 °C for 24 h. After incubation, the diameters of inhibition zones were measured, and isolates were classified as susceptible (S), intermediate (I), or resistant (R) according to the interpretive criteria recommended by EUCAST for *Bacillus* spp. [20]. Additionally, due to the absence of interpretive criteria for certain antibiotics against *Bacillus* spp. in the EUCAST guidelines, the standards established for *Staphylococcus* were applied as reference. *Staphylococcus aureus ATCC 29213* was used as the quality control strain [21].

### 2.4. Detection of ARGs

In this study, DNA was extracted from pre-processed probiotic products by using a TIANAMP Stool DNA Kit (Tiangen Biotech, Beijing, China). The high-throughput quantitative PCR (HT-qPCR) analysis was performed by using a StepOnePlus™ real-time fluorescence quantitative PCR system (Thermo Scientific, Waltham, MA, USA), as previously described [8]. A total of 270 primers were used to detect seven types of ARGs (aminoglycosides, β-lactams, macrolide–lincosamide–streptomycin B (MLSB), fluoroquinolone, chloramphenicol, and amphenicol (FCA), sulfonamides, tetracyclines, and vancomycin), six integron genes (*TNPA-01*, *TNPA-02*, *TNPA-03*, *TNPA-04*, *TNPA-05*, *TNPA-07*) and one transposon gene (*INTI1*). According to a previous reference [15], the relative abundance and fold change in ARGs in samples were calculated based on the following method

∆Ct = Ct (ARG) − Ct (16S rRNA), (1)

Relative abundance = 2^−∆Ct^. (2)

The threshold cycle (Ct) of 40 was the detection limit. ARGs were the target genes, and 16S rRNA was the internal reference gene.

### 2.5. Statistical Analysis

For the heatmap, we separately extracted the fold values of each experimental group and created it by using TreeView software (version 1.2.0). Spearman’s correlation was calculated by using IBM SPSS Statistics (version 25.0) to examine the relationship between ARGs and bacterial communities. The co-occurrence network of ARGs and MGEs was established by using Gephi software (version 0.10.1).

## 3. Results

### 3.1. Microbial Composition, Isolation of Dominant Strains, and Accuracy Assessment of Labeling

**Figure 1:**
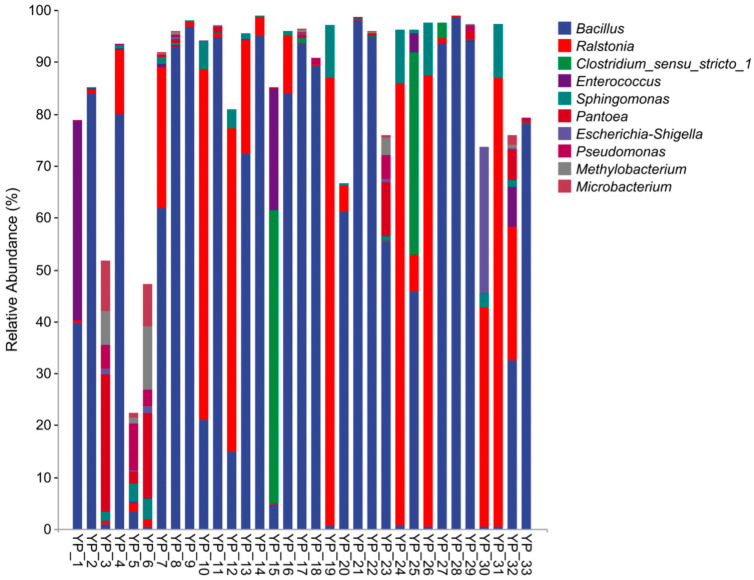
The bacterial community composition of 33 Animal-use commercial probiotic products at the genus levels.

16S rRNA sequencing results revealed that, after denoising, 2,213,506 valid sequences were obtained, with distribution of sequence length ranging from 180 bp to 394 bp, and a total of 1036 amplicon sequence variants (ASVs) were detected. The top 10 genera ranked by abundance at the genus level are *Bacillus*, *Ralstonia*, *Clostridium*, *Enterococcus*, *Sphingomonas*, *Pantoea*, *Escherichia*, *Pseudomonas*, *Methylobacterium* and *Microbacterium* (Figure 1). Sequencing analysis results showed that *Bacillus* spp. existed in all samples, with 19 samples having an abundance higher than 50%. The dominant strain *Bacillus* spp. was isolated and identified, and its morphological characteristics were observed. A total of 32 strains of *Bacillus* spp. were isolated, including 25 strains of *Bacillus subtilis* complex, 4 strains of *Bacillus cereus*, 2 strains of *Bacillus cabrialesii*, and 1 strain of *Bacillus paramycoides* (Table 1).

In this experiment, all 33 probiotic sample labels only specified the species name of the microbial strains, without indicating specific strain names or numbers. Additionally, 11 probiotic product labels did not describe the quantity of viable bacteria in the main ingredients. There were 10 probiotic products with mislabeling problems (Table 1), including failure to isolate the main ingredients as indicated and discrepancies between the isolated strains and the label. Meanwhile, 4 strains of *Bacillus cereus*, 2 strains of *Bacillus cabrialesii*, and 1 strain of *Bacillus paramycoides* isolated in this experiment did not comply with the regulations.

### 3.2. Antimicrobial Resistance of Bacillus spp.

**Figure 2:**
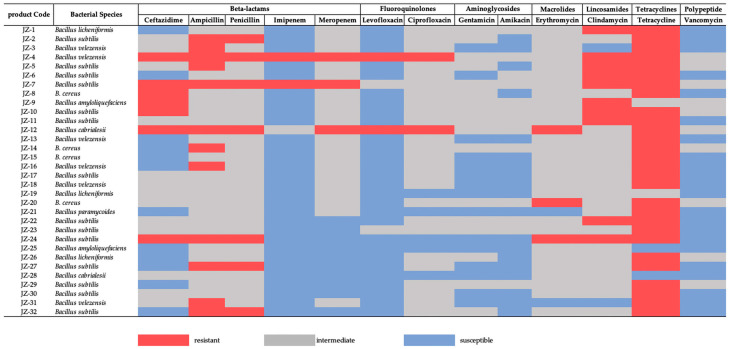
Antibiotic-resistant phenotypes in 32 strains of *Bacillus* spp.

In this study, 13 common antibiotics were selected, and the antibiotic resistance of *Bacillus* spp. were tested using the disk diffusion method. This study demonstrated that some strains exhibited multidrug resistance phenotypes to ampicillin, clindamycin, and tetracycline, while showing marked susceptibility to imipenem, levofloxacin, gentamicin, amikacin, and vancomycin (Figure 2). The antimicrobial susceptibility profiling revealed 87.50% (28/32) tetracycline resistance and 37.50% (12/32) ampicillin resistance in *Bacillus* spp., with all strains remaining susceptible to gentamicin, amikacin and vancomycin (Table S2).

### 3.3. Abundance and Co-Occurrence of ARGs and MGEs

**Figure 3:**
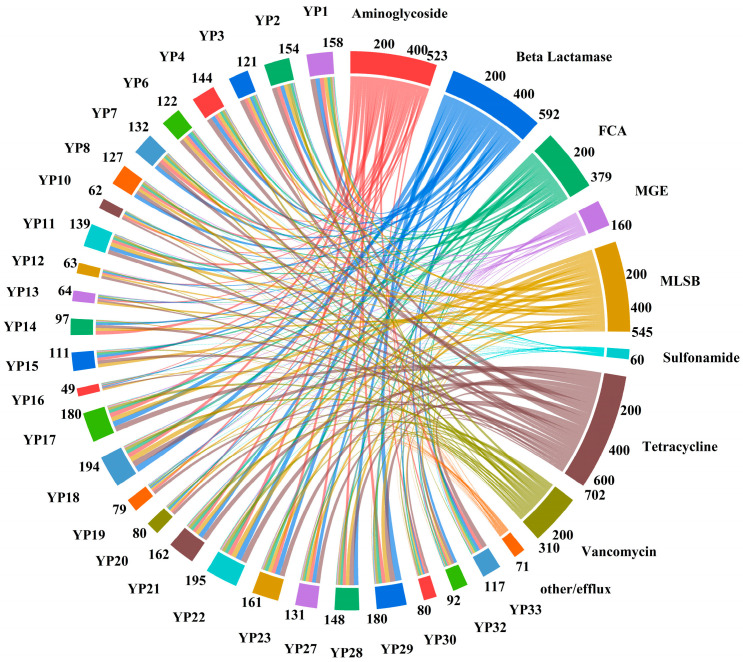
Chord diagram of ARGs in 27 commercial veterinary probiotic products. Sample information is on the left, and ARG information is on the right; the scale represents the abundance of ARGs in each probiotic product.

In this study, ARGs and MGEs were intended to be detected in 27 animal probiotic products with high microbial content, resulting in the identification of 241 ARGs and 7 MGEs (Table S3). The chord diagram vividly illustrates the diversity of ARGs and MGEs in the probiotic samples (Figure 3). Among them, the highest number of ARGs was detected in YP22, reaching 195 species, followed by YP18 with 194 detected ARGs. ARGs had the highest relative abundance in YP30, followed by YP11, and then YP33. The relative abundance in these three samples was nearly 10 times higher than in other samples.

All identified ARGs provide resistance to the 6 major classes of antibiotics, including tetracyclines (21.01%), β-lactams (17.71%), MLSB (16.3%), aminoglycosides (15.65%), FCA (11.34%), and vancomycin (9.28%). Among all probiotic products, the top 10 ARGs with relatively high abundance mainly consisted of β-lactams (*BLATEM*, *BLAOXA1/BLAOXA30*, *AMPC-02*, *AMPC-03*), aminoglycosides (*APHA1*, *AADD*), FCA (*CATA1*, *ACRF*), tetracyclines (*TETL-01*), and MLSB (*ERMK-01*).

**Figure 4:**
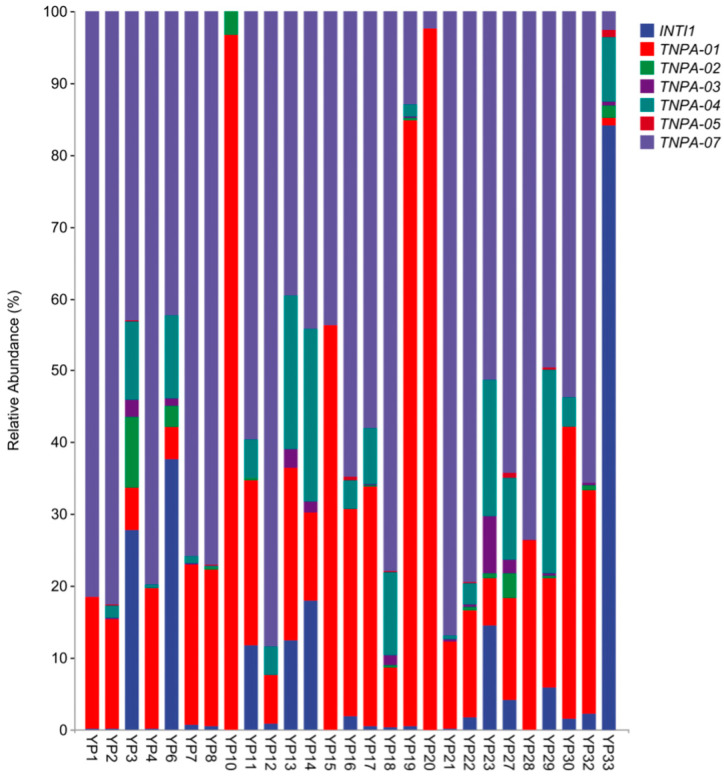
Distribution of MGEs in 27 commercial veterinary probiotic products.

**Figure 5:**
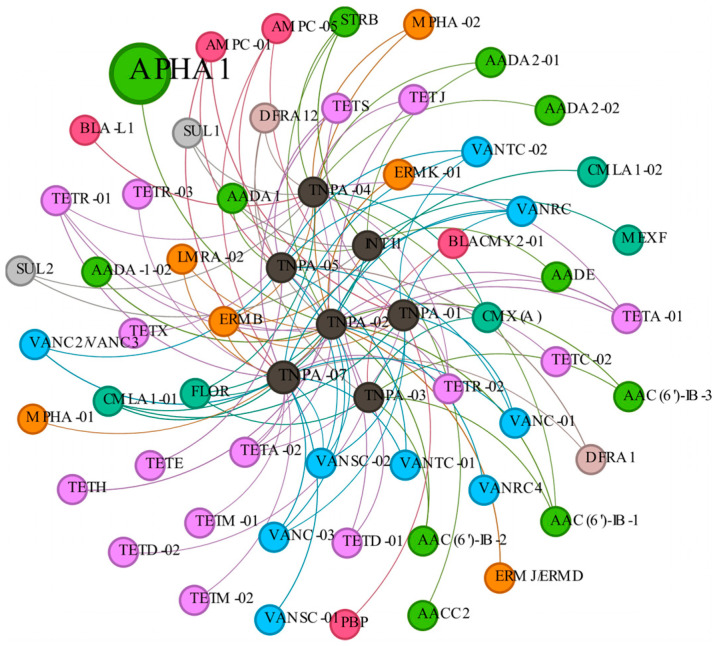
The co-occurrence network of ARGs and MGEs. The node size represents the node degree, and a large size indicates a high degree.

For the detected MGEs in all probiotic products, *TNPA-07* (69.47%) exhibited the highest relative abundance, followed by *TNPA-01* (28.50%), and *TNPA-04* (1.32%) (Figure 4). *TNPA-07* had a high relative abundance which more than 50% in 18 samples. In YP10, YP19, and YP20, the relative abundance of *TNPA-01* was the highest, while in YP33, the relative abundance of *INTI1* was the highest (Figure 4). The co-occurrence network of ARGs and MGEs was visualized using Spearman correlation analysis to understand the relationship between the identified ARGs and MGEs. The co-occurrence network that was created consists of 62 nodes and 114 edges (Figure 5). The 7 central black nodes corresponded to MGEs, and the colored nodes represented ARG subtypes, with edges reflecting their co-occurrence frequencies. In this network, *TNPA-07* and *TNPA-01* were identified as key hubs. The analysis of 7 MGEs showed a positive correlation with ARGs (r > 0.80, *p* < 0.01), particularly *TNPA-07*, *TNPA-02* and *TNPA-01* were extremely significant positive correlation with aminoglycosides, β-lactams, MLSB, FCA, tetracyclines, and vancomycin (r > 0.80, *p* < 0.001). The network analysis revealed statistically significant co-occurrence patterns between several ARGs and MGEs across samples, suggesting potential associations. However, these correlations do not provide direct evidence of ARG–MGE physical linkage or horizontal transfer.

### 3.4. Co-Occurrence of ARGs and Bacterial Community

**Figure 6:**
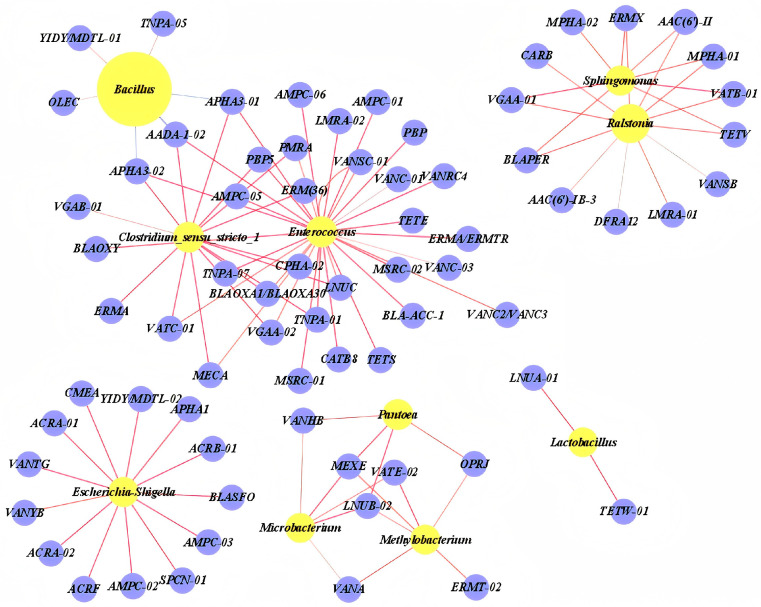
The co-occurrence network of ARGs and Bacterial community. The node size represents the node degree, and a large size indicates a high degree.

The co-occurrence network of ARGs and the bacterial community was visualized using Spearman correlation analysis to understand the relationship between them. The resulting co-occurrence network consisted of 10 nodes and 102 edges (Figure 6). Among the 10 bacterial communities, *Enterococcus*, *Clostridium*, *Escherichia* and *Lactobacillus* were extremely significant positive correlation with multiple ARGs, principally aminoglycosides, β-lactams, FCA and tetracyclines, particularly gene classes conferring resistance to β-lactams, aminoglycosides, MLSB and FCA (r > 0.80, *p* < 0.001). On the other hand, *Enterococcus* showed an extremely significant positive correlation with two MGEs (*TNPA-07* and *TNPA-01*), but no obvious correlation with other ARGs. Furthermore, *Bacillus* showed a weak correlation with three aminoglycosides (*AADA-1-02*, *APHA3-01* and *APHA3-02*), and we also observed that *Bacillus* had the highest abundance in the bacterial community but didn’t show a significant correlation with any ARGs.

## 4. Discussion

### 4.1. Microbial Composition, Isolation of Dominant Strains, and Accuracy Assessment of Labeling

*Bacillus* spp. is a strictly aerobic or facultative anaerobic bacterium capable of producing spores that are highly resistant to adverse conditions. Meanwhile, *Bacillus* spp. can produce antimicrobial substances with a broad spectrum of inhibition, capable of killing bacteria (including drug-resistant strains), certain fungi, parasites, some viruses, and tumor cells [22]. It has been widely used as a major component in probiotic products for aquaculture and animal husbandry to enhance animal performance and treat diseases [9]. Consistent with related research, in this experiment all 33 probiotic sample contained *Bacillus* spp. that commonly added to probiotic products, as they are primarily associated with reducing disease and enhancing animal performance [23,24].

In line with internationally accepted standards for probiotic evaluation, such as the FAO/WHO Guidelines for the Evaluation of Probiotics in Food [25], probiotic microorganisms must be precisely identified to strain level, proven safe for the intended use, and administered in adequate amounts throughout shelf life. In this experiment, all labeling information of all probiotic products is incomplete, 11 probiotic product labels did not describe the quantity of viable bacteria in the main ingredients. According to the “Guidelines for the Identification and Safety Evaluation of Microbial Strains Used in Direct Feeding of Microorganisms and Fermentation Products” issued by the Ministry of Agriculture and Rural Affairs of the People’s Republic of China, microbial strains used in direct feeding must be specified by their genus name, species name (including Chinese name, Latin name, etc.), and strain name or number. Moreover, the quantity of viable bacteria is an important indicator for evaluating the quality of probiotic products. Failure to specify the number of viable bacteria may lead to excessive or insufficient intake by animals, affecting the efficacy and safety of the products.

There were 10 probiotic products with mislabeling problems. It was speculated that there may be contamination in the products, or the manufacturers falsely labeled the main ingredients to achieve profitability [26]. Mislabeling of probiotic products can lead to incorrect dosing or the inclusion of strains that differ from those declared on the label, which may compromise product efficacy and animal health [27]. Moreover, underdosing may fail to achieve adequate colonization or immune modulation, whereas overdosing could increase production costs or lead to digestive imbalance. Inaccurate strain identification may result in the administration of ineffective or even unsafe microorganisms, potentially reducing the expected probiotic benefits or causing unintended microbial interactions in the host. According to Announcement No. 2045 of the Ministry of Agriculture and Rural Affairs of the People’s Republic of China, “Catalog of Feed Additive Varieties (2013)”, *Bacillus* spp. that can be used as feed additives include *Bacillus subtilis*, *Bacillus licheniformis*, *Bacillus lentus*, *Bacillus pumilus*, *Bacillus coagulans*, and *Brevibacillus laterosporus*.

In fact, there are many reasons for contamination of probiotic products, such as defects in quality control during production, improper storage or transportation conditions, and improper staff operation [28]. According to the FAO and WHO safety assessment of commercial probiotic products, there is a significant safety risk associated with the detection of ingredients that are not declared on their labels [25]. A study on 92 probiotic products sourced from the Chinese market, found that 54 strains of non-probiotic bacteria belonging to ESKAPE pathogens (*Enterococcus faecium*, *Staphylococcus aureus*, *Klebsiella pneumoniae*, *Acinetobacter baumannii*, *Pseudomonas aeruginosa*, and *Enterobacter* spp.) were isolated, posing potential harm to animals [8]. The presence of these pathogens in probiotic products greatly contaminates the environment and poses a threat to the health of livestock, poultry, and humans. Research indicate that inaccurate descriptions of active ingredients on probiotic product labels greatly compromise their safety [29]. The *Bacillus cereus* isolated in this study were unlisted components, suggesting contamination of the products with strains. *Bacillus cereus* is an important foodborne pathogen that can cause vomiting and diarrhea in humans and animals [30]. The use of *Bacillus cereus* in probiotics poses significant safety risks, and there are still many deficiencies in the safety assessment and regulation of veterinary probiotics in many regions [31]. The detection of *Bacillus cereus* not only reflects labeling inaccuracies but also reveals potential biosafety risks, highlighting the need to strengthen regulatory oversight of commercial veterinary probiotic products. Therefore, evaluating the accuracy of labels and identifying microbial populations in veterinary probiotics are prerequisites for their safe application. Relevant authorities should formulate more comprehensive policies, and strengthen inspections and regulation of probiotic products, thereby ensuring the safety of commercial probiotic products.

### 4.2. Antimicrobial Resistance of Bacillus spp.

Tetracyclines are broad-spectrum antibiotics whose extensive use in both human and veterinary medicine has driven the widespread emergence of resistance. In probiotic preparations, tetracycline resistance in *Bacillus* spp. is frequently associated with mobile resistance determinants such as *tet(B)* and *tet(45)*, which can be readily transferred between bacteria. [10]. High resistance rates therefore likely reflect past antibiotic use and horizontal gene transfer rather than intrinsic traits of all strains. In European countries, probiotic products containing tetracycline-resistant genes have long been discontinued as feed additives [32]. To mitigate the risk of disseminating tetracycline resistance via veterinary probiotics, China should strengthen regulatory requirements by mandating strain-level identification, routine screening for transferable ARGs, and prohibiting the use of strains that carry mobilizable resistance determinants in feed additives.

Clindamycin belongs to the lincosamides of antibiotics and is the most common drug used to treat Gram-positive bacteria such as *Bacillus* spp. and *Staphylococcus aureus* [33]. In our study, 31.25% (10/32) *Bacillus* spp. isolates exhibited phenotypic resistance to clindamycin. However, correlational research found that among 48 strains of *Bacillus* spp. isolated from probiotic products, were all resistant to clindamycin, with most strains having a minimum inhibitory concentration of 16 μg/mL to clindamycin [28]. The difference from the results of this experiment may be related to the types of probiotic products and the species of *Bacillus* spp. isolated.

In recent years, due to the overuse of antibiotics and the emergence of super-resistant pathogens, there was a growingly number of people concerned that bacterial resistance would pose a threat to global public health. Probiotics, as alternatives to antibiotics, are increasingly being used as feed additives. In addition to studying the potential toxicity of probiotics, attention should also be focused on their antibiotic resistance and the spread of ARGs [34]. Studies showed that multidrug-resistant *Bacillus* spp. were present in most probiotic products, and the emergence of multidrug-resistant bacteria was a significant factor contributing to the widespread occurrence of ARGs in animal faces and the environment [35,36].

### 4.3. Abundance and Co-Occurrence of ARGs and MGEs

From a One Health perspective, the detection of ARGs and MGEs in commercial veterinary probiotics raises significant concerns about potential cross-sectoral transmission of antimicrobial resistance. Probiotic strains carrying ARGs may enter the animal gut microbiome, where HGT could facilitate the spread of resistance determinants to commensal or pathogenic bacteria [37,38]. Once excreted through manure, these bacteria and genetic elements may contaminate the environment—including soil, water, and feed—forming reservoirs that contribute to the persistence and dissemination of resistance across ecosystems. This environmental dissemination not only threatens animal health but also poses potential zoonotic risks through the food chain and agricultural exposure routes.

The study found that there was no correlation between the quantity and relative abundance of ARGs in probiotic samples. Although the number of ARGs detected in YP22 and YP19 was much higher than that in YP30, their abundance was much lower compared to YP30. Moreover, due to differences in production processes and intended applications among different probiotic products, the types of strains contained in the products vary, leading to differences in the subtypes of ARGs being carried. A survey of 561 different probiotic products from 1901 to 2022 showed that the types and proportions of ARGs contained in different probiotic products vary significantly [39].

Studies showed a widespread correlation between the abundance of *INTI1* and ARGs as well as anthropogenic pollutants, with *INTI1* being used as an indicator for assessing anthropogenic contamination [40]. In this experiment, *INTI1* was detected in multiple samples, and some ARGs were closely associated with it. These co-occurring genes may be related to anthropogenic contamination during the production or transportation processes of the products. Furthermore, MGEs play a crucial role in transmitting ARGs to recipient hosts, which leads to the emergence of new antibiotic-resistant bacteria [41], and we noticed that the abundance of MGEs was significantly associated with the detected levels of ARGs. During the manufacturing and storage processes, probiotic strains may acquire ARGs through several mechanisms. One possible route is HGT, facilitated by MGEs such as plasmids, transposons, and bacteriophages that are present in the production environment [14]. Inadequate sterilization or contamination from raw materials can also introduce resistant microorganisms that serve as donors of ARGs. Moreover, prolonged storage under suboptimal conditions may induce stress responses in bacteria, promoting gene exchange and enhancing the persistence of resistant strains. To minimize these risks, strict quality control and biosafety measures should be implemented throughout the production chain. These include using well-characterized and certified probiotic strains, maintaining aseptic manufacturing environments, performing routine screening for ARGs and MGEs, and ensuring proper storage conditions. Establishing standardized guidelines for probiotic production and regular regulatory supervision would further help prevent the acquisition and dissemination of resistance determinants during probiotic formulation and storage.

ARGs may be positively correlated with multiple MGEs, while one type of MGEs may also be associated with multiple ARGs. Based on the present study, the observed ARG–MGE co-occurrences should be interpreted as indicators of possible association rather than proof of transfer. From a One Health perspective, the presence of multidrug-resistant *Bacillus* spp. and MGEs in commercial probiotic formulations underscores the interconnectedness of animal, human, and environmental health. Probiotic strains may act as vectors for antimicrobial resistance dissemination through the food chain and environmental reservoirs.

### 4.4. Co-Occurrence of ARGs and Bacterial Community

Previous research has indicated that environmental factors, particularly antibiotics and heavy metals, along with bacterial communities and MGEs, have been key drivers in shaping the ARGs profile [42]. In probiotic products, microorganisms may carry ARGs and serve as potential hosts for the dissemination of ARGs. The correlation analysis suggested potential ARG hosts when ARGs displayed a robust and statistically significant positive association with co-existing bacterial taxa (r > 0.80, *p* < 0.01) [43]. By comparing these networks, *Clostridium*, *Enterococcus* and *Escherichia* were identified as key hubs, although their abundance was significantly lower than that of *Bacillus*. Moreover, it was not observed that an increased bacterial abundance was significantly associated with the detected ARG levels, which was in stark contrast to the results observed in the dissemination and enrichment of ARGs within the chicken intestinal microbiome [44]. Other studies have shown that the presence of heavy metal residues in soil and water can further contribute to the proliferation and enrichment of drug-resistance genes. Additionally, variations in the microbial community composition could serve as a key factor influencing the abundance of ARGs [45].

These findings collectively demonstrate that the composition of microbial communities in commercial probiotic products. From a One Health perspective, the coexistence of multidrug-resistant *Bacillus* species, ARGs, and MGEs in probiotic formulations highlights the interconnected risks among animal, human, and environmental health systems.

## 5. Conclusions

In this study, we investigated commercial veterinary probiotic products in Northern China. The results revealed issues of mislabeling and bacterial contamination, while *Bacillus* spp. strains exhibited multidrug resistance. ARGs were widely detected in these products and were potentially associated with MGEs, which are key drivers of resistance gene transmission. From a One Health perspective, these findings highlight the potential risks of interspecies ARG transfer and environmental dissemination through manure, feed, and soil. Probiotic genera such as *Bacillus*, *Clostridium*, and *Enterococcus* may act as reservoirs and vectors of ARGs, posing threats not only to animal health but also to human and ecosystem health. Strengthening regulatory oversight and implementing risk management strategies for veterinary probiotics are essential to ensure biosafety and support the sustainable development of animal husbandry.

## Figures and Tables

**Table 1:** Characteristics of the 32 *Bacillus* spp. isolates from 33 commercial probiotic products.

Product ^1^	Source Area	Purpose of Application ^2^	Ingredients on the Label	Isolates
1	Xingtai, Hebei	A, D	*Bacillus licheniformis*	*Bacillus licheniformis*
2	Bayan Nur, Inner Mongolia	A, C, D	*Bacillus subtilis*, *Bacillus licheniformis*	*Bacillus subtilis*, *Bacillus velezensis*,
3	Heilongjiang	B, D	*Saccharomyces cerevisiae*, *Bacillus licheniformis*	None
4	Harbin, Heilongjiang	B, D	*Saccharomyces cerevisiae*, *Bacillus subtilis*	*B* *acillus velezensis*
5	Shijiazhuang, Hebei	A	*B* *acillus subtilis*	*Bacillus subtilis*
6	Heilongjiang	A, B, E	*Lactobacillus acidophilus*, *Bacillus subtilis*	*Bacillus subtilis*
7	Harbin, Heilongjiang	B, D	*Lactobacillus acidophilus*, *Bacillus subtilis*	*Bacillus subtilis*
8	Harbin, Heilongjiang	A, D	*Lactobacillus plantarum*, *Bacillus subtilis Saccharomyces cerevisiae*,	*Bacillus cereus*, *Bacillus amyloliquefaciens*
9	Harbin, Heilongjiang	A, D, E	*Saccharomyces cerevisiae*, *Bacillus subtilis*	None
10	Harbin, Heilongjiang	A, C	*Bacillus subtilis*	*Bacillus subtilis*
11	Harbin, Heilongjiang	A, B, C, D, F	*Lactobacillus acidophilus*, *Bacillus subtilis*	*Bacillus subtilis*
12	Shenzhen, Guangdong	A, B, C, D, E	*Lactic acid bacteria*, *Bacillus licheniformis*	*Bacillus cabrialesii*
13	Harbin, Heilongjiang	A, D	*Lactobacillus acidophilus*, *Bacillus subtilis*, *Saccharomyces cerevisiae*,	*Bacillus velezensis*
14	Harbin, Heilongjiang	A, C	*Lactobacillus acidophilus*, *Bacillus subtilis*	*Bacillus cereus*
15	Pizhou, Jiangsu	A, B, C, D	*Bacillus subtilis*	*Bacillus cereus*
16	Harbin, Heilongjiang	A, B, C	*Bacillus subtilis*, *Yeast*	*Bacillus velezensis*
17	Xianyang, Shaanxi	A, B, E	*Saccharomyces cerevisiae*, *Bacillus subtilis*	*B* *acillus subtilis*
18	Nanning, Guangxi	A, B, E	*Bacillus licheniformis*, *Bacillus subtilis*	*Bacillus velezensis*
19 *	Shandong	A, D	*Lactobacillus acidophilus*, *Bacillus subtilis*	*Bacillus licheniformis*
20	Shandong	A, B, C, E	*Lactobacillus plantarum*, *Bacillus subtilis*	*Bacillus cereus*
21	Shangqiu, Henan	A, D	*Bacillus subtilis*	*Bacillus paramycoides*
22	Fuyang, Anhui	A, B, C, E	*Bacillus subtilis*	*Bacillus subtilis*
23	Xilingol League, Inner Mongolia	C	*Bacillus subtilis*	*Bacillus subtilis*
24	Baoding, Hebei	A, C, D	*Lactobacillus*, *Bifidobacterium*, *Bacillus licheniformis*	*Bacillus subtilis*
25	Dalian, Liaoning	A, B, C	*Clostridium butyricum*, *Bacillus subtilis*	*Bacillus amyloliquefaciens*
26	Weifang, Shandong	A, B, C, D	*Bifidobacterium*, *Bacillus licheniformis* *Streptococcus*	*Bacillus licheniformis*
27	Chengdu, Sichuan	A, B, C, D, E	*Bacillus subtilis*	*Bacillus subtilis*
28	Jinan, Shandong	A, B, E	*Bacillus subtilis*	*Bacillus cabrialesii*
29	Nanchang, Jiangxi	A, B, E	*Bacillus subtilis*, *Lactobacillus plantarum*	*Bacillus subtilis*
30	Shangqiu, Henan	A, B, D	*Bacillus subtilis*	*Bacillus subtilis*
31	Shangqiu, Henan	A, B, E	*Bacillus subtilis*	None
32	Harbin, Heilongjiang	A, B, D	*Bacillus subtilis*	*Bacillus velezensis*
33 *	Shijiazhuang, Hebei	A, B, D	*Bacillus licheniformis*, *Bacillus subtilis*, *Saccharomyces cerevisiae*	*Bacillus subtilis*

* Products marked with an asterisk are in liquid form, while all other products are in powder form. ^1^ 10 of 33 probiotic products were mislabeled or contaminated and indicated in underlined. ^2^ Purposes of probiotic product application: A = regulating the balance of gut microbiota in livestock and poultry; B = improving feed conversion rate; C = enhancing the growth rate of livestock and poultry; D = boosting the resistance of livestock and poultry; E = improving the breeding environment; F = supplementing vitamins.

## Data Availability

The raw data supporting the conclusions of this article will be made available by the authors on request.

## Supplementary Materials

The following supporting information can be downloaded at: https://www.mdpi.com/article/10.3390/biology14111612/s1, Table S1: Standards for interpreting of inhibition zone diameters for antibiotics used in this study; Table S2. Antimicrobial resistance frequencies of *Bacillus* spp. in 32 probiotic products; Table S3: Melting temperature (Tm) values of ARGs detected by high-throughput qPCR.
