# Supplementary material for: Assessment of Antibiotic Resistance and Microbial Contamination in Commercial Veterinary Probiotic Products
Source: Biology (Basel). 2025 Nov 17;14(11):1612. doi: 10.3390/biology14111612 (PMC12649896; doi:10.3390/biology14111612)
Supplement: Supplementary file 1 [file biology-14-01612-s001.zip › biology-3956376-supplementary.pdf]

Assessment of Antibiotic Resistance and Microbial Contamination in Commercial Veterinary Probiotic Products

Table S1. Standards for interpreting of inhibition zone diameters for antibiotics used in this study

Antibiotic	Disk content (μg)	Zone diameter breakpoints (mm)[a]	
		R＜	S≥
Ceftazidime	30	14	20
Ampicillin	10	12	16
Penicillin	10	18	50
Imipenem	10	13	16
Meropenem	10	14	20
Levofloxacin	5	23	50
Ciprofloxacin	5	23	50
Gentamicin	10	18	22
Amikacin	30	15	18
Erythromycin	15	13	18
Clindamycin	2	14	20
Tetracycline	30	14	19
Vancomycin	5	14	17

Note：[a] Ranges of zone of inhibition diameters exhibited by bacteria considered susceptible (S) and resistant (R) to each antibiotic are shown. The Intermediate (I) category is not listed but is interpreted as the values between the S and the R breakpoints. If the S and R breakpoints are the same value, there is no I category.

Table S2. Antimicrobial resistance frequencies of *Bacillus spp*. in 32 probiotic products

Antibiotics	S	I	R	S（%）	R（%）
Ceftazidime	12	13	7	31.50	21.88
Ampicillin	0	20	12	0	37.50
Penicillin	0	25	7	0	21.88
Imipenem	29	1	2	90.63	6.25
Meropenem	10	19	3	31.25	9.38
Levofloxacin	28	2	2	87.50	6.25
Ciprofloxacin	5	25	2	15.62	6.25
Gentamicin	15	17	0	46.88	0
Amikacin	19	13	0	59.38	0
Erythromycin	2	27	3	6.25	9.38
Clindamycin	2	20	10	6.25	31.25

Tetracycline	2	2	28	6.25	87.50
Vancomycin	22	10	0	68.15	0

Note: S, susceptible; I, intermediate; R, resistant. S (%) and R (%) indicate the percentage of isolates classified as susceptible or resistant, respectively.

Table S3 Melting temperature (Tm) values of ARGs detected by high-throughput qPCR.

**No.**	**Gene name**	**Melting temperature (Tm)**
1	*16S rRNA*	81.65711975
2	*AAC(6′)-IB-1*	81.50778198
3	*AAC(6′)-IB-2*	82.40383911
4	*AAC(6′)-IB-3*	82.10514832
5	*AAC(6′)-II*	80.01435089
6	*AACA/APHD*	71.80049133
7	*AACC*	81.51143646
8	*AACC2*	79.26763916
9	*AACC4*	82.70252228
10	*AADA-01*	81.80646515
11	*AADA-02*	82.10514832
12	*AADA1*	78.8196106
13	*AADA-1-01*	79.26821899
14	*AADA-1-02*	76.28131104
15	*AADA2-01*	80.61231995
16	*AADA2-02*	82.70315552
17	*AADA2-03*	80.31362915
18	*AADA5-01*	82.85250092
19	*AADA5-02*	83.74856567
20	*AADA9-01*	82.55381012
21	*AADA9-02*	81.80708313
22	*AADD*	81.06035614
23	*AADE*	71.50227356
24	*ACRA-01*	81.50839233
25	*ACRA-02*	82.55381012
26	*ACRA-03*	86.88673401
27	*ACRA-04*	81.35904694
28	*ACRA-05*	81.50839233
29	*ACRB-01*	81.80958557
30	*ACRF*	82.10830688
31	*ADEA*	80.76405334
32	*AMPC/BLADHA*	83.00448608
33	*AMPC-01*	81.06277466
34	*AMPC-02*	81.36149597
35	*AMPC-03*	81.95894623
36	*AMPC-04*	80.0113678

37	*AMPC-05*	81.35533142
38	*AMPC-06*	84.64058685
39	*AMPC-07*	84.49125671
40	*AMPC-08*	85.53656006
41	*AMPC-09*	86.43814087
42	*APH6IA*	80.91316986
43	*APHA1*	76.72890472
44	*APHA3-01*	76.28076935
45	*APHA3-02*	76.28076935
46	*BLA1*	75.8326416
47	*BLA-ACC-1*	74.93868256
48	*BLACMY*	81.35958099
49	*BLACMY2-01*	81.50895691
50	*BLACMY2-02*	86.7371521
51	*BLACTX-M-01*	85.69151306
52	*BLACTX-M-02*	83.15210724
53	*BLACTX-M-03*	76.72890472
54	*BLACTX-M-04*	81.06276703
55	*BLACTX-M-05*	75.23741913
56	*BLACTX-M-06*	86.58776855
57	*BLAGES*	79.26933289
58	*BLAIMP-01*	77.77935028
59	*BLA-L1*	82.40577698
60	*BLAMOX/BLACMY*	78.9706192
61	*BLAOKP*	86.88641357
62	*BLAOXA1/BLAOXA30*	75.83417511
63	*BLAOXA10-01*	75.68482208
64	*BLAOXA10-02*	78.22384644
65	*BLAOXY*	79.86682129
66	*BLAPAO*	82.70448303
67	*BLAPER*	79.11997986
68	*BLAPSE*	77.92514038
69	*BLAROB*	81.50965118
70	*BLASFO*	85.54222107
71	*BLASHV-01*	85.8418808
72	*BLASHV-02*	85.69252014
73	*BLATEM*	78.82217407
74	*BLATLA*	78.52414703
75	*BLAVEB*	73.89344788
76	*BLAVIM*	87.63304138
77	*BLAZ*	73.29266357
78	*CARB*	81.95645905
79	*CATA1*	78.97153473
80	*CATB3*	83.15348053

81	*CATB8*	79.56895447
82	*CEPA*	77.02991486
83	*CFIA*	83.90026093
84	*CFR*	81.65777588
85	*CFXA*	76.8805542
86	*CMEA*	83.30258179
87	*CMLA1-01*	84.49546814
88	*CMLA1-02*	84.49546814
89	*CMX(A)*	85.2421875
90	*CPHA-01*	88.6765213
91	*CPHA-02*	89.12457275
92	*DFRA1*	78.52177429
93	*DFRA12*	82.10598755
94	*EMRB/QACA*	78.07374573
95	*EREA*	78.96980286
96	*EREB*	79.26848602
97	*ERM(34)*	81.80730438
98	*ERM(36)*	82.85270691
99	*ERMA*	74.63887024
100	*ERMA/ERMTR*	75.08774567
101	*ERMB*	74.93755341
102	*ERMC*	74.63861847
103	*ERMF*	74.04115295
104	*ERMJ/ERMD*	75.98291779
105	*ERMK-01*	76.5803833
106	*ERMK-02*	77.3272171
107	*ERMT-01*	72.69685364
108	*ERMT-02*	71.65238953
109	*ERMX*	83.1525116
110	*ERMY*	79.71611023
111	*FLOR*	82.85377502
112	*FOLA*	81.06137848
113	*FOX5*	86.88667297
114	*INTI1*	90.62083435
115	*LMRA-01*	84.64363098
116	*LMRA-02*	75.98291779
117	*LNUA-01*	73.59305573
118	*LNUB-01*	73.74198914
119	*LNUB-02*	73.89133453
120	*LNUC*	77.17953491
121	*MATA/MEL*	77.62480927
122	*MDTA*	75.23538208
123	*MECA*	74.49224091
124	*MEFA*	80.91026306

125	*MEXA*	84.64058685
126	*MEXD*	88.82181549
127	*MEXE*	83.2966156
128	*MEXF*	84.0432663
129	*MPHA-01*	82.25131226
130	*MPHA-02*	81.50466156
131	*MPHB*	79.71682739
132	*MPHC*	78.21842957
133	*MSRA-01*	75.68741608
134	*MSRA-02*	75.68741608
135	*MSRC-01*	75.98616791
136	*MSRC-02*	77.33056641
137	*NDM1*	86.28474426
138	*OLEC*	81.96125793
139	*OPRJ*	85.24755859
140	*PBP*	77.33056641
141	*PBP2X*	76.88022614
142	*PBP5*	77.03180695
143	*PENA*	81.50984955
144	*PIKR1*	87.47943115
145	*PIKR2*	89.12425232
146	*PMRA*	72.54714203
147	*QNRA*	83.30565643
148	*SPCN-01*	84.79183197
149	*SPCN-02*	71.79862976
150	*STR*	72.39806366
151	*STRA*	80.16195679
152	*STRB*	83.74528503
153	*SUL1*	86.13417816
154	*SUL2*	85.53695679
155	*SULA/FOLP-01*	75.8303833
156	*SULA/FOLP-02*	82.1044693
157	*SULA/FOLP-03*	82.55429077
158	*TET(32)*	80.31125641
159	*TET(36)-01*	78.22097778
160	*TET(36)-02*	78.22097778
161	*TET(37)*	81.95251465
162	*TET(38)*	80.61038208
163	*TETA-01*	82.10077667
164	*TETA-02*	81.95145416
165	*TETB-01*	79.26367188
166	*TETB-02*	79.26367188
167	*TETC-01*	75.53063202
168	*TETC-02*	75.53063202

169	*TETD-01*	82.10077667
170	*TETD-02*	82.10077667
171	*TETE*	80.45823669
172	*TETG-01*	85.53517151
173	*TETG-02*	87.32702637
174	*TETH*	77.6211319
175	*TETJ*	75.67995453
176	*TETK*	71.94691467
177	*TETL-01*	80.75688171
178	*TETL-02*	78.21841431
179	*TETM-01*	75.83402252
180	*TETM-02*	77.4768219
181	*TETO-01*	76.43140411
182	*TETO-02*	78.22355652
183	*TETPA*	74.19122314
184	*TETPB-01*	78.67158508
185	*TETPB-02*	73.44449615
186	*TETPB-03*	76.58074951
187	*TETPB-04*	73.44449615
188	*TETPB-05*	77.1781311
189	*TETQ*	77.1781311
190	*TETR-01*	85.54148102
191	*TETR-02*	85.54148102
192	*TETR-03*	75.83402252
193	*TETS*	73.29515076
194	*TETT*	74.93795013
195	*TETU-01*	78.67066956
196	*TETU-02*	77.62339783
197	*TETV*	81.06126404
198	*TETW-01*	81.06126404
199	*TETX*	75.53510284
200	*TNPA-01*	77.62608337
201	*TNPA-02*	84.79516602
202	*TNPA-03*	85.09387207
203	*TNPA-04*	82.70417786
204	*TNPA-05*	76.58059692
205	*TNPA-07*	77.77544403
206	*VANA*	82.25369263
207	*VANB-01*	79.56771088
208	*VANC-01*	77.62608337
209	*VANC-02*	76.28188324
210	*VANC-03*	81.65852356
211	*VANC1*	77.17802429
212	*VANC2/VANC3*	80.61306763

213	*VANHB*	83.60006714
214	*VANHD*	83.4519577
215	*VANRA-01*	79.71697235
216	*VANRA-02*	79.41353607
217	*VANRB*	81.0565033
218	*VANRC*	75.68088531
219	*VANRC4*	78.21937561
220	*VANRD*	75.97915649
221	*VANSA*	79.11647034
222	*VANSB*	81.80311584
223	*VANSC-01*	80.90674591
224	*VANSC-02*	77.02958679
225	*VANTC-01*	80.01688385
226	*VANTC-02*	79.86751556
227	*VANTE*	76.88021851
228	*VANTG*	82.99794769
229	*VANWG*	77.77641296
230	*VANXA*	72.8453598
231	*VANXB*	75.83389282
232	*VANXD*	80.61183929
233	*VANYB*	81.95862579
234	*VANYD-01*	79.86751556
235	*VANYD-02*	77.1751709
236	*VATB-01*	75.23721313
237	*VATB-02*	73.44483185
238	*VATC-01*	77.02677917
239	*VATE-01*	76.42954254
240	*VATE-02*	78.66918182
241	*VGAA-01*	75.23440552
242	*VGAA-02*	77.02870178
243	*VGAB-01*	70.15726471
244	*VGAB-02*	78.07374573
245	*VGB-01*	82.84984589
246	*VGB-02*	80.90882111
247	*VGBB-01*	74.48851776
248	*YIDY/MDTL-01*	82.25260925
249	*YIDY/MDTL-02*	79.41572571
